# Artificial intelligence in Immuno-genetics

**DOI:** 10.6026/973206300200029

**Published:** 2024-01-31

**Authors:** Raed Farzan

**Affiliations:** 1 Department of Clinical Laboratory Sciences, College of Applied Medical Scienecs, King Saud University, Riyadh - 11433, Saudi Arabia; 2 Center of Excellence in Biotechnology Research, King Saud University, Riyadh - 11433, Saudi Arabia; 3 Medical and Molecular Genetics Research, King Saud University, Riyadh-11433, Saudi Arabia

**Keywords:** Artificial intelligence, immunology, genetics, machine learning, deep learning

## Abstract

Rapid advancements in the field of artificial intelligence (AI) have opened up unprecedented opportunities to revolutionize various scientific domains, including immunology and genetics. Therefore, it is of interest to explore the emerging applications of AI in immunology and genetics, with the objective of enhancing our understanding of the dynamic intricacies of the immune system, disease etiology, and genetic variations. Hence, the use of AI methodologies in immunological and genetic datasets, thereby facilitating the development of innovative approaches in the realms of diagnosis, treatment, and personalized medicine is reviewed.

## Background:

Immunology and genetics are highly intertwined scientific disciplines that intricately contribute to our understanding of the immune system and its genetic underpinnings [1]. The immune system, which serves as the body's defense mechanism against pathogens and abnormal cells, consists of an elaborate network of cells, molecules, and organs that work in unison to protect the host [2]. Genetics, the study of genes and heredity, plays a pivotal role in determining an individual's immune response and susceptibility to diseases. Genes, segments of DNA, contain instructions for building proteins, which are integral to various cellular processes. Genetic variations, such as single nucleotide polymorphisms (SNPs), insertions, and deletions, can influence the functioning of immune-related genes and proteins, affecting an individual's immune response [3]. Understanding the genetic basis of immune-related diseases is essential for personalized medicine and targeted therapies [4]. Therefore, immunology and genetics are intricately linked disciplines that provide insights into the complexity of the immune system and its genetic regulation. The interplay between these fields has significant implications for understanding immune responses, disease susceptibility, and the development of targeted therapies [5,6].

Artificial intelligence (AI) has emerged as a transformative technology, exhibiting tremendous potential to revolutionize the healthcare landscape by augmenting diagnostic accuracy, enabling personalized medicine, optimizing treatment plans, and advancing medical research [7]. AI encompasses a wide range of techniques and algorithms that empower computer systems to emulate human-like intelligence, including perception, reasoning, learning, and decision-making capabilities [7]. By leveraging AI, healthcare stands to benefit from unprecedented advancements and opportunities. In the domain of medical imaging analysis, AI has made substantial strides. AI-driven virtual screening techniques expedite the analysis of vast chemical libraries, thereby identifying potential drug candidates more efficiently during the initial stages of drug discovery [8]. Additionally, AI plays a pivotal role in designing clinical trials, optimizing trial protocols, and identifying appropriate patient cohorts for enrolment, leading to more efficient and cost-effective research endeavors. Moreover, AI has revolutionized the drug discovery and development processes [9]. The integration of AI in healthcare holds tremendous potential to transform the field by enhancing diagnostic precision, enabling personalized medicine, optimizing treatment strategies, and driving breakthroughs in medical research. Through the application of sophisticated AI algorithms and methodologies, healthcare providers and researchers gain access to unprecedented insights from vast healthcare datasets, empowering informed decision-making and improving patient outcomes. While challenges exist, addressing them through rigorous research, collaboration, and adherence to ethical guidelines can pave the way for a future where AI-driven healthcare becomes an integral part of delivering optimal care to individuals worldwide [9].

## AI in Immunology:

## Machine learning algorithms for immunological data analysis:

Machine learning algorithms have revolutionized immunology by enabling the analysis of complex immunological data applied in various research areas, including the analysis of high-dimensional immunological data, prediction of immune responses, biomarker discovery, and analysis of large-scale immunological datasets [10]. In the analysis of high-dimensional immunological data, machine learning techniques like clustering and dimensionality reduction algorithms have been used to identify distinct cell populations and characterize their phenotypes based on marker expression. This approach enhances our knowledge of immune cell subsets and their roles in diseases [10].

Machine learning models have been developed to predict immune responses, such as vaccine efficacy or disease progression, by training algorithms on immunological and clinical data. Support vector machines, random forests, and artificial neural networks are commonly used algorithms for building predictive models that optimize treatment plans for individual patients [11]. Biomarker discovery has been significantly advanced by machine learning algorithms that integrate multi-omics data. Feature selection and classification algorithms aid in identifying robust biomarkers for diagnostics, disease monitoring, and targeted therapy development [12].

Machine learning techniques, including hidden Markov models and sequence alignment algorithms, have been applied to large-scale immunological datasets, such as immune repertoire sequencing data, to uncover patterns and features related to immune responses [13]. Noteworthy machine learning tools in immuno-genetics research include scikit-learn, an open-source Python library with a wide range of algorithms for data pre-processing and model evaluation [14]. Deep learning techniques, such as convolutional neural networks and recurrent neural networks, extract meaningful features from immuno-genetics data [15]. So, Machine learning algorithms have revolutionized immuno-genetics research by analyzing complex data, predicting immune responses, discovering biomarkers, and uncovering the intricacies of immune repertoire sequencing. These advancements contribute to our understanding of immune system dynamics, disease mechanisms, and personalized medicine, ultimately improving diagnostics, treatments, and patient outcomes in immunology.

## Predictive modelling for immune response and disease outcomes:

Predictive modelling for immune response and disease outcomes l relies on the principles of network theory, where complex networks represent the intricate interactions among immune cells and molecules. Analyzing network topology, connectivity patterns, and identifying key nodes enables the prediction of immune response dynamics and their impact on disease progression [15]. Another influential approach is Bayesian modelling, which employs probabilistic inference to estimate the likelihood of various immune response scenarios based on prior knowledge and observed data. By incorporating comprehensive information about immune system parameters and disease-specific factors, Bayesian models provide valuable insights into disease outcomes and guide personalized treatment strategies [16]. By capturing cellular interactions, environmental influences, and molecular signalling pathways, agent-based models simulate complex immunological processes and predict disease outcomes with high granularity These diverse modelling techniques, which go beyond machine learning, contribute to a deep understanding of immune responses and pave the way for more accurate predictions in personalized medicine [17].

## AI-driven drug discovery and vaccine development:

AI-driven drug discovery and vaccine development have emerged as transformative methodologies revolutionizing biomedical research. In drug discovery, AI algorithms analyze extensive datasets encompassing biological and chemical information. Genomic data, protein structures, and molecular interactions undergo meticulous scrutiny to identify potential drug targets, forecast drug efficacy, and optimize drug candidates [18]. Virtual screening techniques powered by AI models efficiently evaluate vast compound libraries, prioritizing promising candidates for experimental validation. Various generative AI models facilitate the rapid generation of novel molecules with desired properties, enabling the exploration of chemical space and expediting the design of new drug candidates [19]. Within vaccine development, AI algorithms play a pivotal role in antigen identification and design. By harnessing machine learning, comprehensive analysis of pathogen genomic data predicts potential antigens capable of eliciting robust immune responses. This aids in selecting antigenic targets for vaccine development. AI methodologies also optimize vaccine formulations by determining the most effective adjuvants and delivery systems to enhance immune responses [20].

## AI-based analysis of high-dimensional immunological datasets:

AI-based analysis of high-dimensional immunological datasets has emerged as a powerful and transformative approach in unravelling the intricate dynamics of the immune system and gaining novel insights into immune responses. These datasets encompass a wide range of measurements, including gene expression profiles, protein levels, cell populations, and immune cell receptor repertoires, generating complex and heterogeneous data. To extract meaningful information from these datasets, advanced machine learning algorithms such as deep learning, support vector machines, and random forests are utilized. These algorithms enable efficient navigation and analysis of the high-dimensional data, revealing hidden patterns and relationships within the immune system [21].

Dimensionality reduction techniques, such as principal component analysis, t-distributed stochastic neighbour embedding, and manifold learning algorithms, play a crucial role in visualizing and clustering immune cell populations based on their multidimensional features, aiding in the identification of distinct cell subsets and understanding their functional states [22]. Supervised learning models, such as support vector machines, neural networks, and decision trees, enable the prediction of disease outcomes, therapeutic responses, and immune cell functions by learning patterns and associations from labeled data. Unsupervised learning algorithms, including clustering algorithms such as k-means, hierarchical clustering, and density-based clustering, facilitate the characterization of heterogeneity within immune cell populations and the uncovering of distinct functional states [23].

Network-based approaches, such as graphical models, Bayesian networks, and network analysis techniques, contribute to unravelling the complex interactions and communication networks within the immune system, identifying key players, signalling pathways, and regulatory mechanisms governing immune responses [24]. Deep learning architectures, such as convolutional neural networks and recurrent neural networks, excel at extracting features from images, enabling tasks such as cell segmentation, classification, and localization within histological samples [25].

## AI Applications in Genetics:

## Genomic data analysis using AI techniques:

Genomic data analysis using artificial intelligence (AI) techniques has brought about a paradigm shift in the field of genetics, facilitating a comprehensive exploration and interpretation of intricate genomic datasets [26]. Advanced machines learning algorithms, including convolutional neural networks (CNNs), recurrent neural networks (RNNs), and random forests, have been harnessed to uncover latent patterns and extract valuable insights from vast repositories of genomic information [26]. Dimensionality reduction methods such as principal component analysis (PCA), t-distributed stochastic neighbour embedding (t-SNE), and auto-encoders have been effectively employed to reduce the dimensionality of high-dimensional genomic data, enabling visualization, clustering, and the identification of distinct subgroups within the dataset [27]. Clustering algorithms such as k-means, hierarchical clustering, and density-based spatial clustering of applications with noise (DBSCAN) have played a pivotal role in discerning genetic variations and uncovering potential associations with diseases [28]. Classification algorithms like support vector machines (SVMs), decision trees, and Bayesian networks have further augmented the analysis, facilitating tasks such as phenotype prediction, identification of disease markers, and classification of diverse genetic conditions [29]. The integration of these AI techniques into genomic data analysis has not only propelled our comprehension of the genetic underpinnings of diseases but also holds tremendous promise for advancing personalized medicine and driving the development of tailored therapeutic interventions [30].

## Predictive modelling for genetic diseases and susceptibility:

Within this field, an array of machine learning algorithms has been employed, including decision trees, logistic regression, and Bayesian networks. These algorithms have demonstrated their capability to process vast genomic datasets encompassing single nucleotide polymorphisms (SNPs), copy number variations (CNVs), and gene expression profiles, enabling accurate predictions of disease outcomes. To identify the most informative genetic markers associated with disease susceptibility, feature selection methods such as mutual information, genetic algorithms, and sequential forward selection have proven effective [5]. Ensemble methods such as bagging, boosting, and stacking amalgamate multiple predictive models, enhancing predictive performance and capturing intricate relationships between genetic variants and diseases. Augmenting these models with domain knowledge, biological pathway information, and functional annotations further heightens their predictive prowess. Through the utilization of predictive modelling, clinicians and researchers can effectively identify individuals at elevated risk of genetic diseases, enabling proactive intervention, tailored screening protocols, and focused preventive strategies [31].

## AI-driven precision medicine and personalized treatment:

AI-driven precision medicine and personalized treatment have revolutionized healthcare by enabling tailored interventions based on individual patient characteristics and genetic profiles. Cutting-edge tools such as ensemble learning, Bayesian networks, and deep reinforcement learning are applied to analyze extensive datasets containing genomic information, clinical data, and patient outcomes. These tools can uncover intricate patterns, correlations, and predictive markers that contribute to treatment recommendation systems and facilitate drug repurposing efforts [32]. Explainable AI (XAI) techniques like SHapley Additive exPlanations (SHAP) and LIME (Local Interpretable Model-agnostic Explanations) provide valuable insights into the decision-making process of AI models, enhancing interpretability and facilitating the integration of AI-driven solutions into clinical practice [33]. Recent advancements in natural language processing (NLP) have facilitated the integration of electronic health records (EHR) and unstructured patient data, enabling comprehensive analysis and the development of personalized treatment strategies [34]. The integration of diverse AI tools, XAI techniques, and NLP approaches holds great promise for advancing precision medicine, optimizing treatment outcomes, and enhancing patient care in various healthcare settings [35].

## Identification of disease-associated genetic variants:

The field of genetics research has witnessed a revolutionary transformation with the advent of AI-driven approaches for identifying disease-associated genetic variants. By harnessing advanced algorithms and tools, machine learning techniques and deep learning models have enabled researchers to unravel the intricate interplay between genetic variations and susceptibility to various diseases [36]. Variant prioritization algorithms like CADD (Combined Annotation-Dependent Depletion) and VEP (Variant Effect Predictor) play a vital role by integrating diverse functional annotations and genomic features, allowing for the prioritization of genetic variants based on their potential impact on disease development [37]. These AI-driven methodologies not only facilitate the unravelling of the genetic underpinnings of complex diseases but also hold immense promise in guiding the development of targeted therapies and driving the progress of precision medicine initiatives [38].

## Challenges and Limitations of Artificial Intelligence in Immuno-genetics:

The application of Artificial Intelligence (AI) in immuno-genetics, a field dedicated to studying the immune system and its responses, presents various challenges and limitations. While AI has the potential to revolutionize immuno-genetics by enabling faster and more accurate analysis of complex immunological data, certain factors need to be addressed to fully exploit its capabilities. This article will explore the challenges and limitations related to data quality, standardization, and interoperability; interpretability of AI models; ethical considerations and regulatory challenges; and the integration of AI with clinical workflows and decision-making processes [39].

## Data quality, standardization, and interoperability:

Given the heterogeneous nature of immunological data obtained from diverse sources, such as electronic health records, imaging data, genomic data, and proteomic data, integrating and harmonizing these data types while ensuring high data quality becomes a major obstacle. Issues such as lack of standardized data formats, missing values, and inconsistencies can impede the development and performance of AI models. Additionally, ensuring interoperability between different data systems and platforms is crucial to facilitate seamless data sharing and collaboration [40].

## Interpretability of AI models:

In immuno-genetics, where patient health and well-being are of utmost importance, the interpretability of AI models is essential. Deep learning neural networks and other black-box AI models often lack interpretability, making it challenging to comprehend the underlying rationale behind their predictions [41]. The ability to interpret AI model decisions becomes crucial for gaining the trust of healthcare professionals and patients. Developing AI models that can provide explanations for their decisions by highlighting relevant features and biomarkers is therefore crucial for their adoption in clinical settings [42].

## Ethical considerations and regulatory challenges:

The increasing adoption of AI in immuno-genetics raises ethical considerations and regulatory challenges. Issues such as data privacy, consent, bias, and algorithmic fairness need to be carefully addressed to ensure the responsible and ethical use of AI in healthcare [43]. Moreover, regulatory bodies face the challenge of keeping pace with the rapidly evolving AI landscape and adapting regulations and guidelines accordingly. Striking the right balance between innovation and patient safety is of utmost importance in this domain [44].

## Integration of AI with clinical workflows and decision-making processes:

For AI to reach its full potential in immuno-genetics, seamless integration with existing clinical workflows and decision-making processes is essential [45]. This integration requires careful consideration of factors such as data accessibility, compatibility with existing workflows, user interface design, and clinician engagement. Ensuring that AI tools fit into the current healthcare infrastructure and complement the expertise of healthcare professionals is paramount. Additionally, proper training and education for clinicians are necessary to promote effective utilization of AI models and prevent potential pitfalls, such as overreliance or misinterpretation of AI-generated results [46].

## Recent publications exploring AI in diverse field of immuno-genetics:

AI is witnessing rapid expansion within the diverse field of immuno-genetics, as numerous studies leverage machine learning, deep learning, and other AI methods to analyze intricate immuno-genetic data, enhance predictions related to the immune system, develop innovative algorithms for epitope discovery, optimize immunotherapy approaches, and facilitate the identification of novel immunological targets. These developments underscore the increasing significance of AI in advancing our understanding and applications in the field of immuno-genetics. A few such studies are depicted in Table 1 and Table 2.

## Conclusion and Future directions:

The incorporation of AI has resulted in substantial advances in immunology and genetics, radically altering our understanding of delicate biological processes and presenting enormous possibilities for disease detection, treatment, and personalized medicine. Table 2 shows recent algorithmic approaches employed in immuno-genetics have played a crucial role in driving this progress. AI-driven approaches enable the analysis of large-scale clinical and epidemiological data. Sophisticated algorithms, including natural language processing and data mining techniques, identify patterns, risk factors, and predictors of vaccine efficacy and safety. This knowledge informs the development of targeted vaccination strategies and facilitates prediction of vaccine performance in diverse populations.

The deep learning-based methods leveraging artificial neural networks have emerged as powerful tools for the analysis of extensive genomic datasets. By harnessing these algorithms, researchers have been able to unveil hidden patterns and correlations that often remain undetectable through traditional methodologies. In parallel, other machine learning techniques such as random forests and support vector machines have proven highly valuable in predicting the immunogenicity of therapeutic proteins and identifying potential targets for immunotherapies. This information is critical in drug development as it helps identify potential risks associated with immunogenicity and guides the design of safer and more efficacious therapeutic interventions. This facilitates the exploration of intricate relationships between genetic variations and immune responses, yielding novel insights into the mechanisms underlying autoimmune disorders, cancer immunotherapy, and vaccine design. AI algorithms have also been employed to analyze high-dimensional single-cell sequencing data, enabling the identification and characterization of rare immune cell populations. By utilizing unsupervised learning techniques, these algorithms effectively cluster cells based on their gene expression profiles, providing valuable insights into the heterogeneity of immune cell populations and their significance in maintaining health and contributing to disease progression. So, the integration of AI in immuno-genetics has ushered in a new era of scientific exploration, enabling researchers to uncover hidden patterns, make accurate predictions, and accelerate advancements in our understanding of the immune system. These applications demonstrate the immense potential of AI in driving discoveries and innovations in immuno-genetics, ultimately paving the way for improved disease management and the development of tailored therapeutic interventions.

## Figures and Tables

**Table 1:** Description of few recently published studies exploring artificial intelligence in Immunogenetics

**Study Title**	**Reference no**	**Description**
"Deep learning for population genetic inference"	[47]	Investigated the use of deep learning algorithms to infer population genetic parameters from genomic data.
"Using deep learning to model the hierarchical structure and function of a cell"	[48]	Applied deep neural networks to predict genetic interactions, which are crucial for understanding complex diseases.
"Generative adversarial networks and its applications in biomedical informatics"	[49]	Exploring the applications of Generative Adversarial Networks (GANs) ,utilization of Cycle-GAN, a specific variant of GAN, in the field of digital image processing.
"A knowledge graph to interpret clinical proteomics data"	[50]	Clinical Knowledge Graph (CKG) It serves as a comprehensive repository, incorporating pertinent experimental data, information from public databases, and relevant literature.
"Deep learning-based identification of cancer or normal tissue using gene expression data"	[51]	Used deep learning to address the extent to which the machine can learn to recognize cancer.
"Artificial intelligence enables comprehensive genome interpretation and nomination of candidate diagnoses for rare genetic diseases"	[52]	Evaluate the diagnostic efficacy of Fabric GEM, an innovative clinical decision support tool that employs artificial intelligence, aimed at expediting the interpretation of genomes.
"Artificial intelligence applied in neoantigen identification facilitates personalized cancer immunotherapy"	[53]	AI-based platforms for neoantigen prediction address limitations of bioinformatics methods, improve accuracy through scoring systems, and hold promise for advancing neoantigen-based immunotherapy.
"Artificial Intelligence-Assisted Transcriptomic Analysis to Advance Cancer Immunotherapy"	[54]	The integration of artificial intelligence (AI) technology in transcriptomic analysis enables immunotherapy toxicity, therapeutic response prediction, offering insights for cancer immunotherapy research and addressing its challenges.

**Table 2:** Case studies of recently used AI models in the field of immuno-genetics

**Study**	**Findings**	**Reference**
"DeepNetBim: deep learning model for predicting HLA-epitope interactions based on network analysis by harnessing binding and immunogenicity information"	the combination of its binding model with immunogenic models holds potential for broader clinical applications.	[55]
"DeepImmuno: Deep learning-empowered prediction and generation of immunogenic peptides for T cell immunity"	accurately predicting immunogenic peptides, identifying crucial residues for T cell antigen recognition	[56]
"IEPAPI: a method for immune epitope prediction by incorporating antigen presentation and immunogenicity"	AI, effectively integrates antigen presentation and immunogenicity to improve precision in predicting HLA-I binding and T-cell immune responses, valuable tool for T-cell vaccine design in cancer immunotherapy.	[57]
"A transformer-based model to predict peptide-HLA class I binding and optimize mutated peptides for vaccine design"	The TransMut framework, comprising TransPHLA for pHLA binding prediction and the AOMP program for optimizing mutated peptides, demonstrates performance in predicting pHLA binding, enabling the automatic generation of potential peptide vaccines for vaccine design and screening, filling a crucial gap in computational peptide and HLA binding optimization.	[58]
"3pHLA-score improves structure-based peptide-HLA binding affinity prediction."	3pHLA-score surpasses commonly used scoring functions in structural virtual screening, enhancing structure-based methods for epitope discovery and offering potential advancements in cancer and viral vaccine development.	[59]
"High-throughput, targeted MHC class I immunopeptidomics using a functional genetics screening platform"	EpiScan Predictor, a MHC class I peptide binding prediction algorithm, demonstrates comparable performance to existing methods while overcoming the underrepresentation challenge of cysteine-containing peptides, enabling accelerated CD8+ T cell epitope discovery and immunotherapeutics development.	[60]
"MachineTFBS: Motif-based method to predict transcription factor binding sites with first-best models from machine learning library"	Model provides high-affinity transcription factor binding sites in Saccharomyces cerevisiae promoters using high-throughput experimental TF binding data, achieving an impressive average Matthews Correlation Coefficient score of 0.873.	[61]
"DeepCpG: accurate prediction of single-cell DNA methylation states"	Introduced DeepCpG, an AI-based model for accurate prediction of DNA methylation states in single cells.	[62]
